# Intraoperative cholangiography during open cholecystectomy in 73 dogs with benign gallbladder disease

**DOI:** 10.1111/vsu.70157

**Published:** 2026-09-09

**Authors:** Ryusei Konno, Yasuyuki Kaneko, Misaki Matsunaga, Tatsuyuki Osuga, Ryota Okadera, Shidow Torisu, Shushi Yamamoto, Kiyokazu Naganobu

**Affiliations:** ^1^ Faculty of Agriculture, Veterinary Medical Teaching Hospital University of Miyazaki Miyazaki Japan; ^2^ Faculty of Agriculture, Veterinary Internal Medicine University of Miyazaki Miyazaki Japan; ^3^ Laboratory of Companion Animal Surgery, Department of Companion Animal Clinical Sciences, School of Veterinary Medicine Rakuno Gakuen University Ebetsu Japan

## Abstract

**Objective:**

To evaluate intraoperative cholangiography (IOC) during open cholecystectomy in dogs with benign gallbladder disease, including cases with preoperative common bile duct (CBD) obstruction, and to describe the short‐term outcomes.

**Study design:**

Retrospective case series.

**Animals:**

A total of 73 client‐owned dogs.

**Methods:**

Medical records (2014–2022) were reviewed. All dogs underwent open cholecystectomy, bile duct flushing (BDF) with saline, and IOC under subtraction fluoroscopy. Cholangiograms were assessed for complete CBD obstruction (no duodenal passage), partial obstruction (CBD filling defects indicating retained debris), and filling defects in hepatic ducts. Perioperative mortality (≤14 days) and post‐discharge findings during 3 months of follow‐up were recorded.

**Results:**

IOC was evaluable in 72 of 73 dogs; assessment was precluded by catheter perforation in one dog. Complete CBD obstruction was identified in 12.5% (9/72). Partial obstruction with retained debris occurred in 20.8% (15/72); debris migrated to transiently obstruct the distal CBD during contrast injection in four of 15. Among the 66 dogs in which IOC opacified the right and left hepatic ducts, seven (10.6%) had filling defects in the hepatic ducts. One dog developed suspected contrast‐associated shock that resolved with treatment. Perioperative mortality was 6.8% (5/73) in the total cohort; 0% (0/29) in dogs without jaundice, and 11.4% (5/44) in dogs with jaundice. Among 61 dogs with follow‐up, no deaths occurred within 3 months.

**Conclusion:**

During open cholecystectomy, IOC frequently identified retained debris and other biliary abnormalities.

**Clinical significance:**

IOC may be a useful adjunct to BDF when CBD or hepatic duct obstruction or retained debris is suspected. Routine IOC may improve surgical outcomes, particularly in dogs with preoperative CBD obstruction.

## 1 INTRODUCTION

Benign gallbladder diseases in dogs include gallbladder mucocele, biliary sludge, cholelithiasis, and cholecystitis. These conditions can lead to extrahepatic biliary obstruction or gallbladder rupture, either of which may be fatal. Currently, surgical intervention is considered to offer more favorable outcomes than medical treatment,^1^ and cholecystectomy is the standard approach.

The reported mortality rate for canine cholecystectomy, including both elective and non‐elective cases, ranges from 6.0% to 41.7%,^2^, ^3^, ^4^, ^5^, ^6^, ^7^, ^8^, ^9^, ^10^ which is considerably higher than that in humans (0.15%–0.4%).^11^, ^12^ In both dogs and humans, bile duct flushing (BDF) with saline has been described during cholecystectomy.^13^, ^14^, ^15^, ^16^ However, BDF alone may not always be sufficient to confirm complete clearance of intraductal material from the common bile duct (CBD). However, intraductal material (retained debris) may remain in the common bile duct (CBD) despite BDF. In human medicine, retained debris has been associated with postoperative complications such as abdominal and back pain and, in some cases, CBD obstruction.^17^, ^18^ Moreover, BDF does not necessarily exclude partial obstruction, and intraoperative cholangiography (IOC) may be required to accurately assess strictures, small calculi, masses, or other debris.^19^, ^20^, ^21^ In human medicine, since its first description in 1932, IOC has been widely adopted,^21^ enabling fluoroscopic evaluation of the biliary tract and identification of retained debris or bile duct injuries. One study identified retained debris in 11.6% of cholecystectomy cases,^18^ and IOC is reportedly associated with fewer perioperative complications.^18^, ^22^

While BDF during cholecystectomy is reported in the veterinary field,^14^, ^15^ only one study has described IOC in dogs.^16^ This previous study identified retained CBD material in only 2.1% of cases. However, the authors used a laparoscopic approach and included only cases without preoperative CBD obstruction based on their cases selection criteria. These restrictions do not reflect current clinical practice in which cholecystectomy in dogs is predominantly performed via laparotomy, and many dogs with indications for cholecystectomy have preoperative CBD obstruction.

We hypothesized that performing open cholecystectomy with IOC, including in dogs with preoperative CBD obstruction, would reveal retained debris in a greater proportion of cases. Therefore, we evaluated the clinical utility of IOC in open cholecystectomy.

## 2 MATERIALS AND METHODS

### 2.1 Study population

This retrospective study included dogs diagnosed with benign gallbladder diseases (biliary sludge, gallbladder mucocele, or cholelithiasis) at the University of Miyazaki between May 2014 and June 2022. The inclusion criteria were dogs with benign gallbladder diseases that underwent open cholecystectomy along with BDF and IOC during the study period. No minimum follow‐up period was required for inclusion in this retrospective study. The exclusion criteria were malignancy and a dog with a history of permanent tracheostomy. Information regarding clinical signs, preoperative blood test results, diagnostic imaging, medical history, comorbidities, including endocrinopathies and cardiovascular diseases, hospitalization records, surgical records, and postoperative course was collected from electronic or paper medical records. Additionally, data related to surgery were obtained from intraoperative external camera recordings and surgical logbooks.

### 2.2 Preoperative evaluation

Bodyweight and age were recorded at the time of admission prior to surgery. As preoperative screening, all dogs underwent blood testing, thoracic and abdominal radiography, and abdominal ultrasonography. Gallbladder volume was measured by ultrasound using the formula *V* = 0.53 × length × width × height and was then normalized to bodyweight (mL/kg). The reference range for gallbladder volume was 0.6 (0.4–1.9) mL/kg.^23^ The presence of CBD dilation was also assessed, with a CBD diameter ≥3.5 mm defined as dilated.^24^

Blood tests included measurements of the serum concentrations of blood urea nitrogen, creatinine, alanine aminotransferase, aspartate aminotransferase, alkaline phosphatase, gamma‐glutamyl transferase, total cholesterol, triglycerides, glucose, total protein, albumin, total bilirubin (T‐bil), lipase activity (v‐LIP assay; FUJI DRI‐CHEM, Japan), and C‐reactive protein, and the white blood cell count. Some parameters were not measured in all cases. Preoperative jaundice was defined as T‐bil ≥0.5 mg/dL. If T‐bil was not available, preoperative jaundice was assessed based on mucous membrane color as described in the medical record and an icterus index ≥4, based on our institutional criteria with reference to the reported canine reference interval.^25^ Dogs showing jaundice together with ultrasonographic findings suggestive of biliary obstruction, including CBD dilation and/or gallbladder rupture, within 3 months prior to presentation were considered to have preoperative CBD obstruction. In jaundiced dogs, the medical records were reviewed for evidence of other major causes of hyperbilirubinemia, and no clear evidence of overt hemolysis or other primary non‐obstructive causes was identified.

The diagnosis of benign gallbladder disease was based on preoperative ultrasonographic findings, intraoperative characteristics of gallbladder contents, and histopathologic examination.

### 2.3 Anesthesia

Atropine (0.04 mg/kg) was administered 15–30 min before anesthetic induction. Anesthesia was induced with IV alfaxalone (1–3 mg/kg) or propofol (1–5 mg/kg) until endotracheal intubation was possible. Anesthesia was maintained with isoflurane or sevoflurane. Immediately after induction, methylprednisolone (5 mg/kg) was administered as part of the institution's routine perioperative management. Fentanyl (20–42 μg/kg/h) was continuously infused for intraoperative analgesia. Cefmetazole sodium (25 mg/kg) was administered as a prophylactic antibiotic.

Before laparotomy, a catheter (22 or 24 gauge) was placed in the dorsal pedal or femoral artery and connected to a pressure transducer to allow direct arterial blood pressure monitoring in all dogs. Hypotension (mean arterial pressure < 60 mmHg) was recorded when it persisted for more than 5 min during the procedure.

### 2.4 Open cholecystectomy, IOC, and evaluation methods

Each surgery was performed by one of four veterinarians. Surgery was performed via a standard ventral midline laparotomy with the dog positioned in dorsal recumbency. After laparotomy, two stay sutures (2‐0 or 3‐0 absorbable monofilament) were placed in the gallbladder body, and a 1‐cm incision was made with a scalpel blade. The gallbladder contents were sampled for bacterial culture, followed by decompression of remaining contents by suction. The incision was closed using the stay sutures. An Eagle hook (Atom Vets Medical Co., Ltd., Kyoto, Japan) was used to pass a braided nylon suture (2‐0 or 3‐0) around the cystic duct, and a 10–15‐cm segment of catheter (6 Fr feeding tube) was used as a tourniquet for temporary ligation. A small serosal incision was made on the gallbladder with electrocautery, and the subserosal (fibrous) layer was dissected to separate the gallbladder from the liver. The gallbladder neck was ligated with a braided nylon suture, and Metzenbaum scissors were used to transect the cystic duct between this ligature and the tourniquet. The tourniquet was loosened, a catheter (4–7 Fr feeding tube) was inserted into the duct, and the tourniquet was re‐tightened (Video S1). Non‐contrast fluoroscopic images were obtained before contrast administration in all dogs. IOC was then performed by injecting undiluted non‐ionic contrast (iohexol) under C‐arm fluoroscopy (OEC9900 Elite, GE Healthcare, Tokyo, Japan). The C‐arm was set to vascular subtraction mode at 15 frames/s, and contrast was injected during a brief apneic pause. The intended dose was 2 mL/kg, with the injection terminated when the full dose was reached or when retrograde opacification extended to the distal hepatic ducts. Fluoroscopic images were evaluated by two veterinarians (YK and MM) for the presence of CBD obstruction, CBD filling defects, and filling defects in the right and left hepatic ducts. Complete CBD obstruction was defined as the absence of contrast passage into the duodenum, while CBD filling defects were interpreted as partial obstruction. Filling defects within the CBD or hepatic ducts were considered to indicate retained debris.

In cases with complete or partial CBD obstruction, BDF was performed by slowly injecting saline through the catheter. BDF and IOC were repeated until the obstruction was relieved or filling defects were no longer observed. When complete patency could not be achieved, one or both of the following procedures were performed: the tourniquet was loosened and saline was injected to hydraulically express intraductal contents in a retrograde manner through the cystic duct stump; and/or a duodenotomy with wedge enlargement of the major duodenal papilla was performed to facilitate passage of obstructive material that could not be cleared through the papillary outlet, followed by repeat BDF to achieve antegrade clearance of debris. Thus, retained debris was removed either by antegrade passage into the duodenum or by retrograde expulsion through the cystic duct stump. IOC findings were used to guide the need for additional intraoperative procedures to restore biliary patency.

After IOC, the cystic duct was permanently ligated with 3‐0 nylon suture, and the mucosal surface at the gallbladder neck was cauterized with bipolar forceps. Liver biopsy was performed in all dogs using biopsy forceps (Karl Storz SE & Co. KG, Tuttlingen, Germany) or the guillotine method, with samples collected from multiple lobes. The abdominal cavity was lavaged with saline, and the abdomen was closed in a routine manner. The resected gallbladders and liver specimens were submitted for histopathologic evaluation.

### 2.5 Postoperative management

Postoperatively, cefmetazole (25 mg/kg) was administered. Dogs with gallbladder rupture or suspected infection additionally received empirical meropenem (13 mg/kg) in selected high‐risk cases, such as those with suspected septic shock or bile peritonitis. Antimicrobial therapy was subsequently adjusted according to susceptibility results. If postoperative testing showed increased serum lipase activity, compatible clinical signs, and/or ultrasonographic findings suggestive of pancreatitis (Aplio 400; Canon Medical Systems, Japan), medical management for pancreatitis was instituted. Ultrasonographic examinations were performed by veterinarians with at least 5 years of clinical experience.

### 2.6 Postoperative course

The perioperative mortality rate was defined as the proportion of dogs that died within 2 weeks after surgery. Among cases with available post‐discharge follow‐up, mortality within 3 months was also assessed. Mortality was also calculated separately for dogs with and without preoperative jaundice. During follow‐up, the presence of postoperative pancreatitis and an increased serum T‐bil concentration was evaluated. An increase in T‐bil was defined as either a postoperative value higher than the preoperative value or a rebound increase after an initial postoperative decline from a preoperative high value.

### 2.7 Statistical analysis

Associations of postoperative hyperbilirubinemia and perioperative death with postoperative pancreatitis, intraoperative hypotension, and duodenal papillotomy were evaluated using Fisher's exact test. Preoperative CRP concentrations were compared between dogs with and without preoperative jaundice and between dogs with and without postoperative hyperbilirubinemia using the Wilcoxon rank‐sum test. A value of *p* < .05 was considered statistically significant. Statistical analysis was performed using JMP Pro 17 (SAS, Cary, North Carolina).

## 3 RESULTS

### 3.1 Preoperative findings

A total of 73 dogs were evaluated. The median age was 10.75 years (interquartile range [IQR]: 9.08–12.33), and the median bodyweight was 3.65 kg (IQR: 2.44–6.66). There were 40 females (32 spayed) and 33 males (21 castrated). The study population comprised Chihuahua (*n* = 19), Toy Poodle (*n* = 13), Pomeranian (*n* = 7), Miniature Dachshund (*n* = 5), mixed‐breed dog (*n* = 5), Yorkshire Terrier (*n* = 4), Shetland Sheepdog (*n* = 3), Shiba (*n* = 3), Shih Tzu (*n* = 2), Pug (*n* = 2), Bichon Frise (*n* = 2), Maltese (*n* = 2), Miniature Schnauzer (*n* = 2), American Cocker Spaniel (*n* = 1), Cairn Terrier (*n* = 1), Wire Fox Terrier (*n* = 1), and French Bulldog (*n* = 1). Clinical signs included lethargy (*n* = 58), anorexia (*n* = 52), vomiting (*n* = 52), diarrhea (*n* = 14), polydipsia (*n* = 10), polyuria (*n* = 9), and tachypnea (*n* = 1). Nine dogs were asymptomatic. Recorded comorbidities included endocrinopathies in 16 dogs (hypothyroidism, *n* = 9; hyperadrenocorticism, *n* = 6; diabetes mellitus, *n* = 1) and cardiovascular diseases in nine dogs (mitral regurgitation and/or tricuspid regurgitation). Preoperative blood testing revealed elevations in liver enzymes and T‐bil (Table 1). Jaundice, suspected to be obstructive in origin, was present in 44 dogs.

**TABLE 1:** Preoperative blood test results.

Variable	Median (IQR)	Number of cases with missing data	Reference interval
BUN (mg/dL)	13.6 (11.2–19.9)	5	9.2–29.2
Creatinine (mg/dL)	0.55 (0.41–0.80)	4	0.40–1.40
ALT (U/L)	498 (158–989)	0	17–78
AST (U/L)	80 (40–160)	1	17–44
ALP (U/L)	1029 (238–3306)	0	≤ 89
GGT (U/L)	56 (16–151)	6	≤ 14
Total cholesterol (mg/dL)	346 (230–440)	12	115–337
Triglycerides (mg/dL)	112 (76–260)	25	23–149
Glucose (mg/dL]	107 (96–116)	9	75–128
Total protein (g/dL)	6.7 (6.4–7.2)	33	5.0–7.2
Albumin (g/dL)	3.1 (2.8–3.4)	12	2.6–4.0
Lipase (U/L)	120 (63–270)	21	10–160
Total bilirubin (mg/dL)	1.6 (0.6–4.7)	8	≤ 0.5
CRP (mg/dL)	3.7 (1.0–10.9)	0	≤ 0.7
WBC (×10^4^/μL)	1.47 (0.83–2.43)	0	0.60–1.70
Hematocrit (%)	43 (37–47)	0	37–55

Abbreviations: ALP, serum alkaline phosphatase concentration; ALT, serum alanine aminotransferase concentration; AST, serum aspartate aminotransferase concentration; BUN, serum blood urea nitrogen concentration; CRP, serum C‐reactive protein concentration; IQR, interquartile range; GGT, serum gamma‐glutamyltransferase concentration; WBC, white blood cell count.

The median preoperative CRP concentration in the overall cohort was 3.7 mg/dL (IQR: 1.0–10.9 mg/dL). Preoperative CRP concentration was significantly higher in dogs with preoperative jaundice than in those without preoperative jaundice (median, 7.5 mg/dL [IQR: 2.9–17.5 mg/dL] vs. 0.9 mg/dL [IQR: 0.6–2.1 mg/dL]; *p* < .001). In contrast, preoperative CRP concentration did not differ significantly between dogs with and without postoperative hyperbilirubinemia (median, 1.3 mg/dL [IQR: 0.6–7.5 mg/dL] vs. 3.9 mg/dL [IQR: 1.1–12.9 mg/dL]; *p* = .145).

On ultrasonography, CBD dilation was identified in 34 of 62 dogs (54.8%) with available measurements. The median gallbladder volume to bodyweight ratio was 3.85 mL/kg (IQR: 2.12–6.19). Based on findings recorded within 3 months prior to presentation, including prior jaundice and ultrasonographic evidence suggestive of biliary obstruction, 56 dogs were judged to have preoperative CBD obstruction.

### 3.2 IOC

IOC was performed in all 73 dogs. Cholangiographic evaluation was successful in 72 dogs, while catheter perforation of the bile duct precluded assessment in one dog. In that dog, the cystic duct was ligated distal to the perforation site. Immediately after contrast administration, one dog developed shock with a precipitous drop in blood pressure; clinical signs improved following administration of epinephrine and an antihistamine.

Among the 72 evaluable dogs, complete CBD obstruction was identified in nine dogs (12.5%) (Figure 1). Partial CBD obstruction with intraluminal filling defects, interpreted as retained debris, was identified in 15 dogs (20.8%) (Figure 2). In four of the 15 dogs with retained debris, the retained material migrated during contrast injection and transiently obstructed the distal CBD.

**FIGURE 1:**
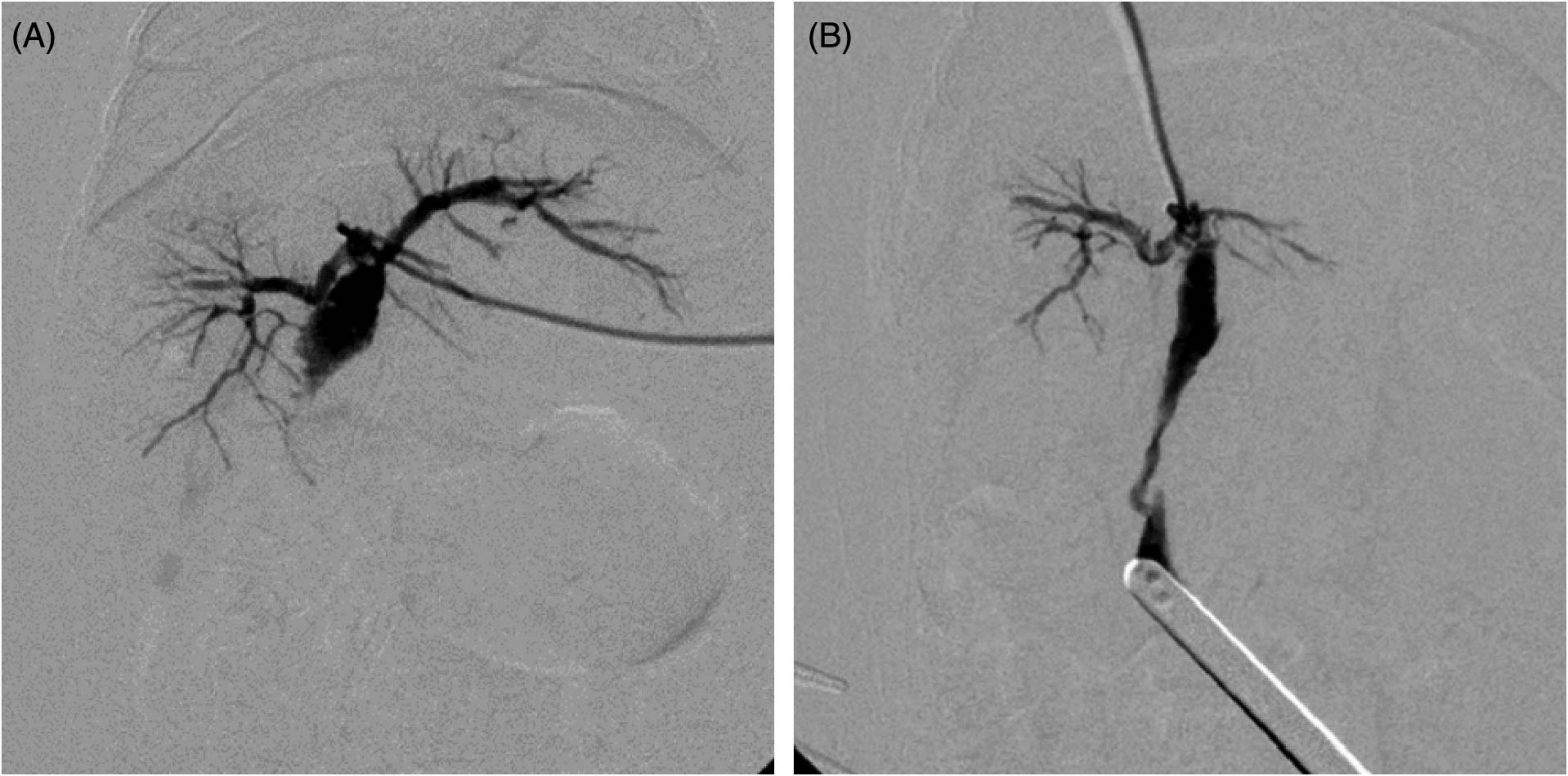
Complete common bile duct obstruction on intraoperative cholangiography. (A) Image obtained before relief of obstruction showing no passage of contrast into the duodenum. (B) Image obtained after relief of obstruction showing contrast freely entering the duodenum, indicating restored patency.

**FIGURE 2:**
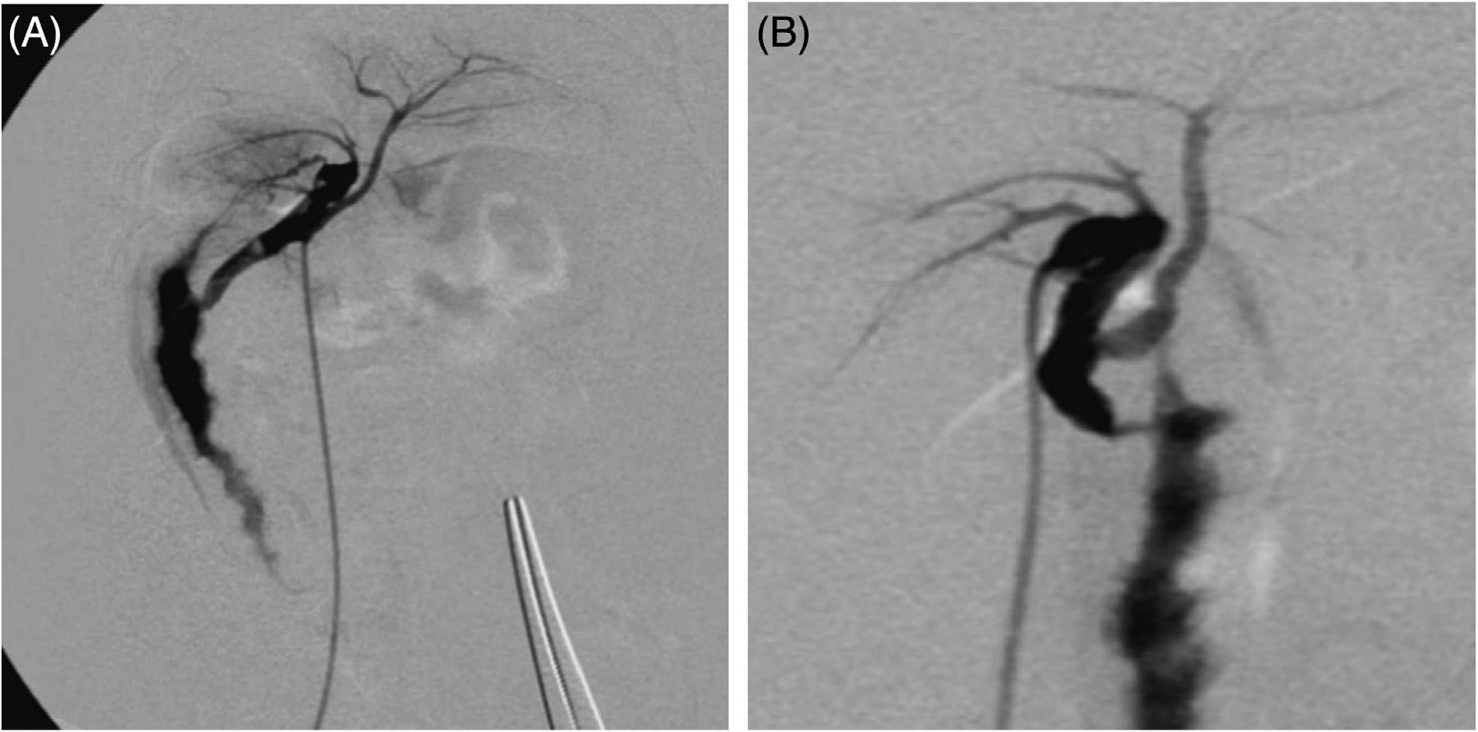
Partial common bile duct obstruction on intraoperative cholangiography. (A) Intraluminal filling defects are visible within the common bile duct. (B) After bile duct flushing, the defects are no longer evident.

Duodenal papillotomy was performed in nine dogs (12.3%) overall when complete biliary patency could not be achieved by antegrade flushing alone. This procedure was intended to relieve complete obstruction in seven dogs and to relieve partial obstruction in two dogs. In one of the two dogs with partial obstruction, preoperative ultrasonography demonstrated a 6–7‐mm intraductal structure; therefore, papillotomy was performed prior to cholecystectomy to extract the material. In the other dog, partially obstructive debris migrated during IOC to occlude the CBD, necessitating papillotomy to restore patency.

A stenotic configuration of the CBD without frank obstruction was observed in one dog (Figure 3); the cause was unknown. In 47 dogs (65.3%), neither complete nor partial CBD obstruction was detected, contrast passed into the duodenum, and no stenotic appearance was observed. Filling defects within the right and/or left hepatic ducts were observed in seven of 66 dogs (10.6%) in which the hepatic ducts were opacified (Figure 4). These defects were identified in the left hepatic duct near the junction with the CBD and at a branch confluence in one dog, in the left hepatic duct after branch convergence but before entry into the CBD in two dogs, in the right hepatic duct after branch convergence but before entry into the CBD in two dogs, and bilaterally after branch convergence but before entry into the CBD in two dogs.

**FIGURE 3:**
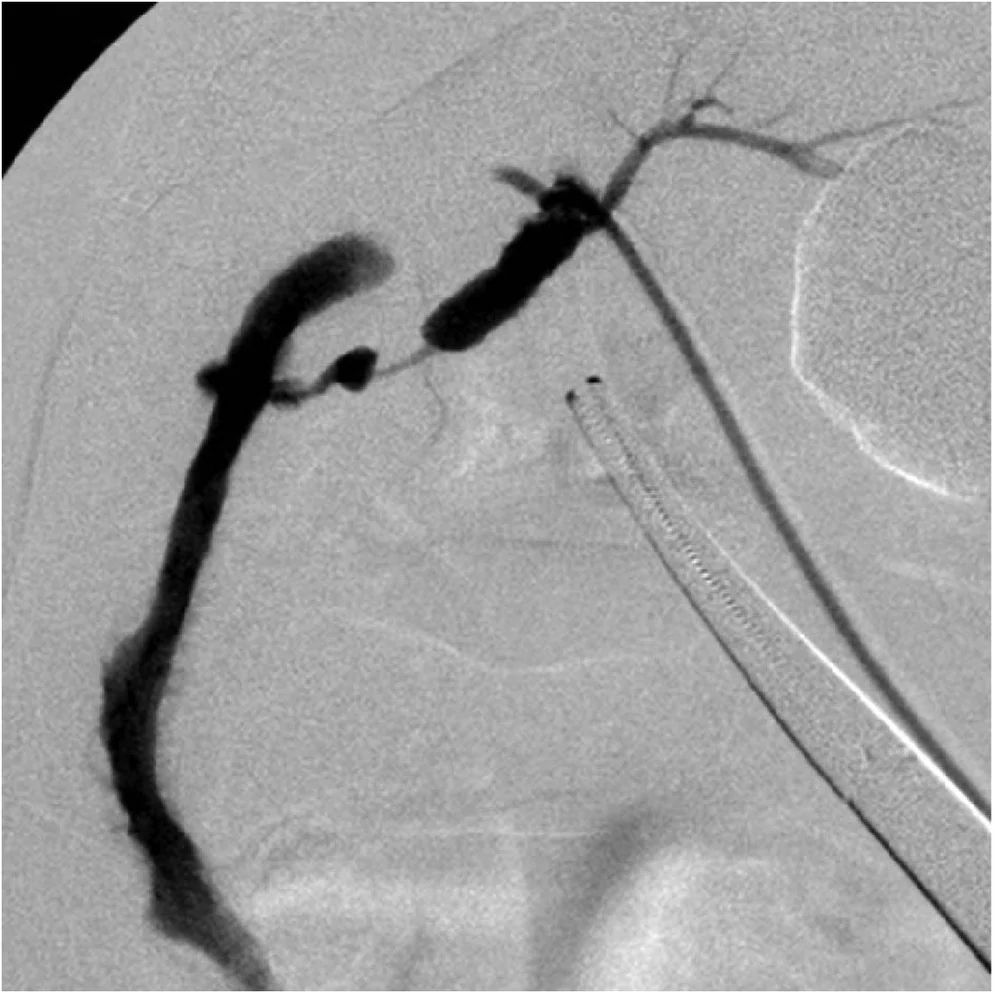
Suspected segmental common bile duct stenosis on intraoperative cholangiography. The common bile duct exhibits mid‐duct narrowing, subsequent dilatation, and a second distal narrowing.

**FIGURE 4:**
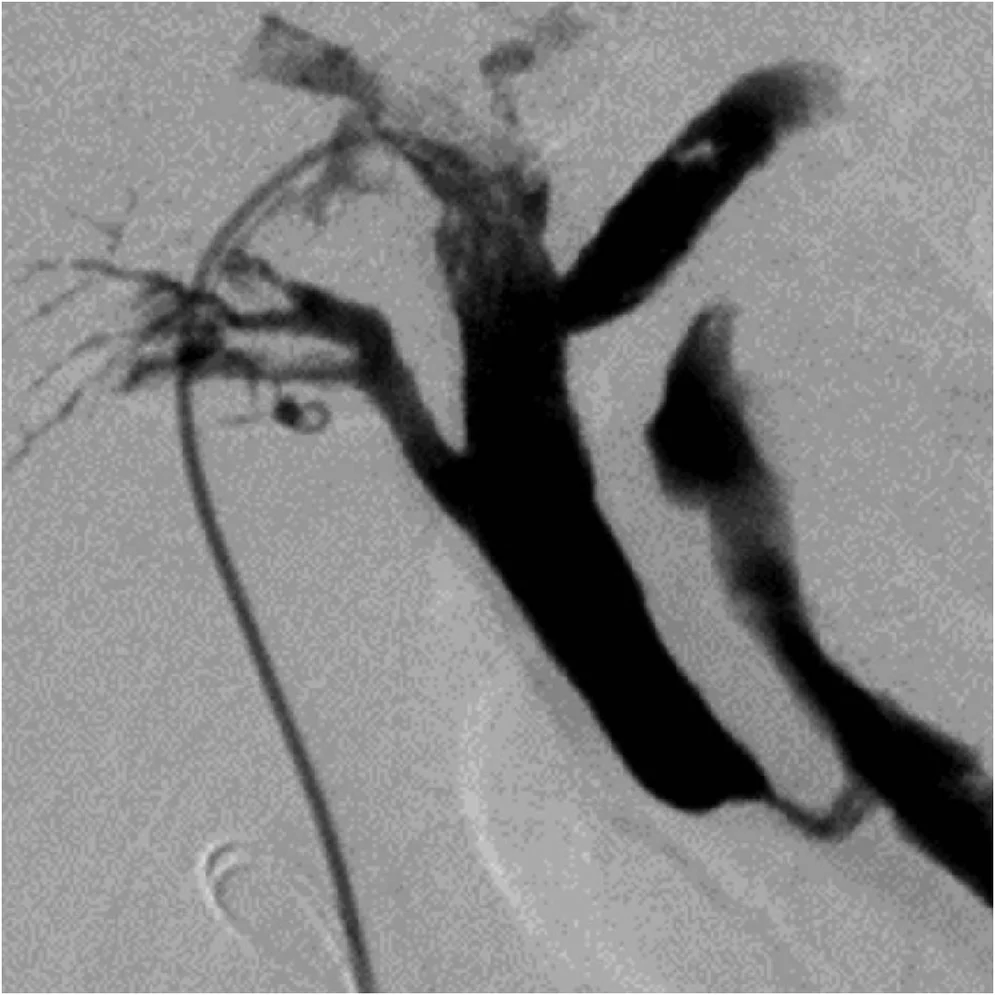
Filling defects within the hepatic ducts on intraoperative cholangiography.

Bacterial culture of gallbladder contents was performed in 68 dogs, and bacterial growth was detected in 15 dogs (22.1%).

### 3.3 Histopathologic diagnoses

Histopathologic evaluation of the gallbladder was available for 67 dogs. Because the pathology reports were not fully standardized across cases, the gallbladder histopathologic findings were diverse and not mutually exclusive. The most commonly described findings included cholecystitis in 35 dogs (52.2%), gallbladder necrosis in 25 dogs (37.3%), and mucosal calcification in seven dogs (10.4%). Less common findings included mucosal hyperplasia in four dogs (6.0%), wall thinning in three dogs (4.5%), ulceration in two dogs (3.0%), gallbladder myositis in one dog (1.5%), and no remarkable histopathologic change in three dogs (4.5%).

Liver biopsy was performed in all 73 dogs, revealing hepatitis in 41 dogs (56.2%), fibrosis in 16 (21.9%), and hepatic abscesses in six (8.2%). In two of the six dogs with hepatic abscesses, IOC demonstrated punctate, poorly defined opacities within the hepatic parenchyma (Figure 5); this appearance was not observed in other dogs.

**FIGURE 5:**
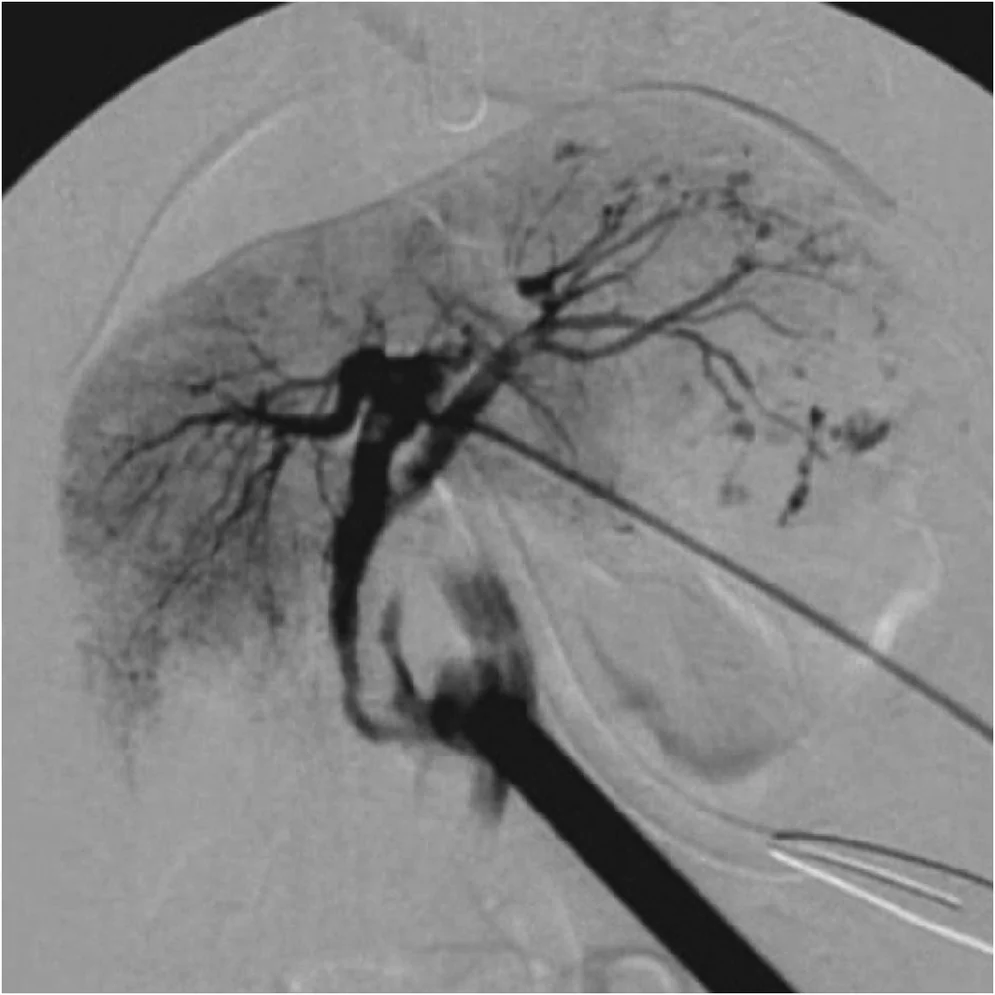
Intrahepatic bile duct disruption on intraoperative cholangiography in a dog with histopathologically confirmed hepatic abscessation. Multiple punctate, ill‐defined opacities are present within the hepatic parenchyma.

### 3.4 Postoperative course

Five dogs (6.8%) died within 2 weeks after surgery. The suspected causes of death were acute kidney injury followed by euthanasia immediately after surgery, suspected pulmonary thromboembolism on postoperative day (POD) 2, status epilepticus of unknown cause on POD 9, presumed acute hepatic dysfunction on POD 1, and an undetermined cause on POD 10. The relationship of these deaths to IOC or BDF remained uncertain. When stratified by preoperative jaundice, the mortality rate was 0% (0/29) in dogs without jaundice and 11.3% (5/44) in dogs with jaundice. Among the 61 dogs with available post‐discharge follow‐up, no deaths occurred within 3 months.

Postoperative elevation of the serum lipase activity together with compatible clinical signs and/or ultrasonographic findings suggestive of pancreatitis was observed in 13 dogs (excluding those with a preoperatively increased serum lipase activity); these abnormalities improved with medical management in all 13 dogs.

A postoperative increase in serum T‐bil concentration was identified in eight dogs. Based on postoperative ultrasonographic findings and clinicopathologic results, the cause was considered likely extrahepatic obstructive in three dogs, likely hepatic in three dogs, and undetermined in two dogs. Of the three dogs with presumed extrahepatic obstruction, two had postoperative CBD dilation on POD 4 and improved with medical management, including trepibutone. In the remaining dog, increased serum bilirubin concentration occurred concurrently with findings suggestive of pancreatitis on POD 5, and pancreatitis‐associated CBD obstruction was suspected; this dog also improved with medical management. In the three dogs with presumed hepatic jaundice, no clear evidence of hepatic failure had been recognized preoperatively. One dog developed increased serum T‐bil concentration approximately 1 month after surgery without evidence of CBD obstruction or hemolysis; recent vaccination had been administered, although its relationship was unclear, and histopathologic examination of the liver showed no remarkable changes. Another dog had hypoalbuminemia (1.7 g/dL) and hypoglycemia (64 mg/dL) without evidence of extrahepatic obstruction or hemolysis on POD 1, and hepatic jaundice was suspected; histopathologic examination identified chronic portal hepatitis. In the remaining dog, hepatic jaundice was suspected based on coagulopathy (PT, 8.8 s; APTT, 100.5 s; antithrombin, 65.7%), hypoalbuminemia (1.1 g/dL), and hyperammonemia (138 μg/dL) on POD 1, and histopathologic examination detected chronic cholangiohepatitis with marked cholestasis.

Postoperative hyperbilirubinemia was associated with duodenal papillotomy (*p* = .006), but not with postoperative pancreatitis (*p* = 1.000) or intraoperative hypotension (*p* = .341). Perioperative death was associated with intraoperative hypotension (*p* = .020) and duodenal papillotomy (*p* = .012), but not with postoperative pancreatitis (*p* = 1.000). The incidence of postoperative pancreatitis did not differ between dogs with and without duodenal papillotomy (3/9 vs. 10/64, *p* = .194).

The dog with a stenotic configuration of the CBD without frank obstruction did not develop death or obstructive jaundice during the 3‐month follow‐up period, although pre‐existing liver enzyme elevations persisted.

## 4 DISCUSSION

In the present study, we performed IOC during open cholecystectomy in dogs, including those with preoperative CBD obstruction. Among the 72 dogs that underwent successful cholangiographic assessment, complete CBD obstruction was identified at the time of BDF and/or the initial cholangiogram in nine dogs. Among these nine dogs, one dog had no preoperative evidence of CBD obstruction, no jaundice, and no CBD dilation, suggesting that intraluminal material within the gallbladder or bile ducts precipitated obstruction during the short interval between preoperative evaluation and intraoperative imaging.

Among dogs with retained debris, four developed distal CBD obstruction during IOC due to migration of the intraductal material. These observations indicate that retained debris can precipitate CBD obstruction and therefore should be removed whenever possible. The residual obstruction identified by IOC was removed using BDF, and the final cholangiograms confirmed complete clearance of the retained material, indicating that BDF can remove retained debris from the CBD. This suggests that routine IOC may help reduce postoperative common bile duct obstruction after cholecystectomy. Although migration of retained debris during IOC may be considered a potential disadvantage of the procedure, the resulting obstruction can be recognized immediately on cholangiography. This allows prompt intraoperative management and may be preferable to an unrecognized postoperative obstruction. Notably, some dogs with preoperative jaundice did not show complete or partial CBD obstruction on IOC. This suggests that preoperative jaundice did not necessarily reflect a persistent fixed obstruction at the time of surgery. Possible explanations include spontaneous passage of intraductal material during the short interval between preoperative evaluation and surgery, or displacement of obstructive material during contrast injection, such that no clear filling defect was visible on cholangiography.

A prior report on IOC in dogs excluded cases with preoperative CBD obstruction.^16^ As hypothesized, retained debris was identified more frequently in the present study than in that report. This difference may be explained by two factors. First, our study included dogs with preoperative CBD obstruction, indicating greater disease severity than in the previous cohort. Accordingly, intraductal gallbladder contents may have been more likely to be present at the time of surgery. Because retained debris can persist despite BDF, IOC may be useful to avoid overlooking such material. Second, the previous study used fluoroscopic images acquired without subtraction, which may have made retained debris more difficult to detect because of superimposed shadows from bone, viscera, and metal. By using digital subtraction fluoroscopy, we minimized the background opacity to more clearly observe the contrast flow, which may have reduced the frequency of missed defects. Because retained debris may be present and may persist despite BDF, performing IOC is useful to avoid missed intraductal material; moreover, the use of subtraction imaging may further reduce such oversights.

All dogs in this study underwent open cholecystectomy using subserosal dissection. Therefore, the effect of this technique on surgical outcome or on the performance of IOC could not be assessed. Although subserosal dissection may have facilitated exposure of the cystic duct and catheter placement, this was not formally evaluated. Likewise, because all cases underwent catheterization, the present study does not allow conclusions regarding whether routine catheterization is necessary in all dogs undergoing cholecystectomy. However, based on our findings, catheterization with IOC may be useful for identifying CBD obstruction and confirming complete removal of retained debris. In addition, duodenal papillotomy was performed as an additional procedure when complete relief of CBD obstruction could not be achieved, and it was associated with postoperative hyperbilirubinemia and perioperative death. Although the influence of the procedure itself cannot be excluded, these findings may also reflect the greater severity of disease in dogs that required papillotomy.

In the present study, the perioperative mortality rate was 6.8% in the overall cohort, 0% in dogs without preoperative jaundice, and 11.4% in those with preoperative jaundice. In a previous report investigating mortality after cholecystectomy, the mortality rate was 9% in the overall cohort, 2% in the non‐jaundiced group, and 20% in the jaundiced group.^5^ These observations suggest that confirming retained debris with IOC and addressing it appropriately may improve the surgical outcomes in canine cholecystectomy. Additionally, in a subset of dogs that were histopathologically diagnosed with hepatic abscesses, IOC demonstrated punctate, poorly marginated opacities within the hepatic parenchyma. Such findings were not observed in dogs without hepatic abscesses. In human medicine, cholangiography has also been reported to be useful for detecting abnormalities associated with hepatic abscesses.^26^ These findings suggest that IOC may provide ancillary information relevant to the detection of hepatic abscesses, although further investigation in larger case series is warranted.

Among the 66 dogs in which IOC opacified the right and left hepatic ducts, there were seven dogs with filling defects interpreted as intrahepatic retained material, three of which subsequently developed a postoperative increase in the serum T‐bil concentration. These findings suggest that debris within the hepatic ducts may migrate into the CBD and precipitate obstruction. Supporting this theory, ultrasonography performed at the time of postoperative serum T‐bil concentration increase in one of the three dogs demonstrated echogenic intraluminal material within the CBD (Figure 6). Therefore, dogs exhibiting hepatic duct filling defects on IOC warrant close postoperative monitoring for signs of CBD obstruction. Further work is needed to establish effective intraoperative strategies to clear intrahepatic duct debris.

**FIGURE 6:**
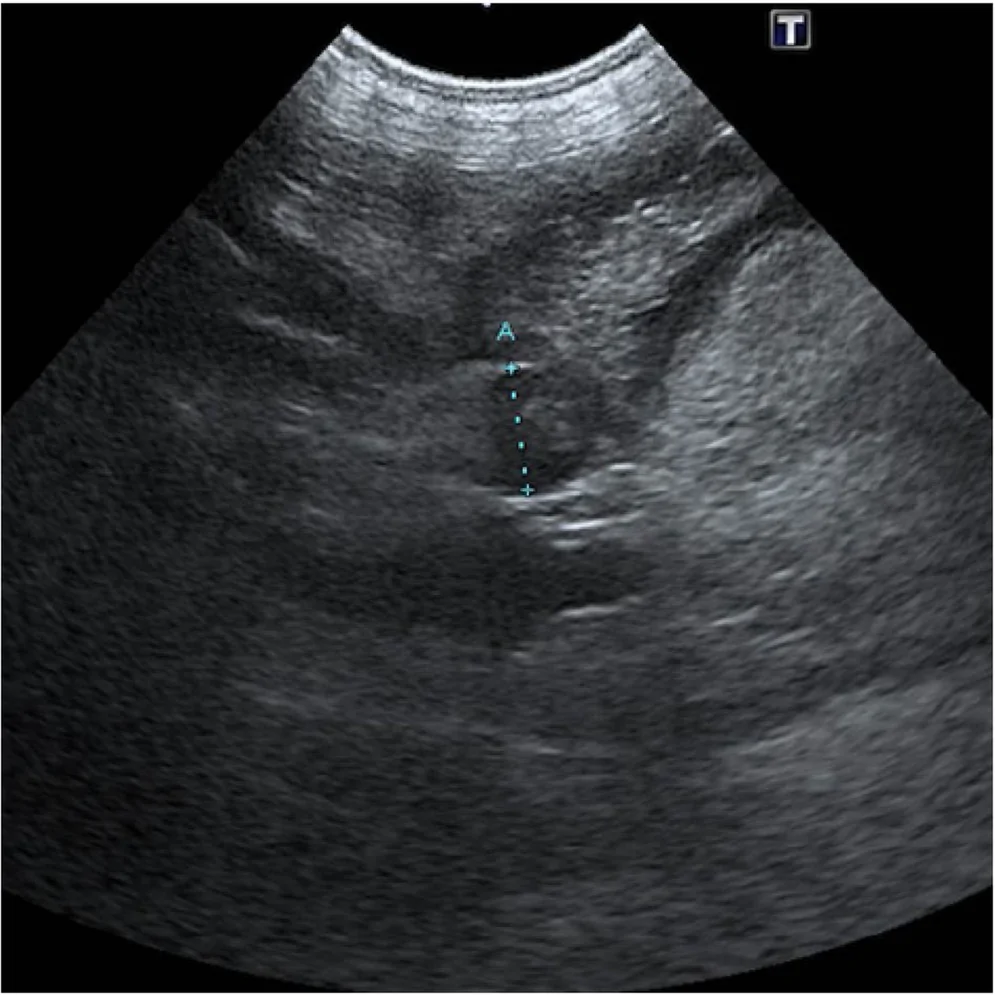
Ultrasonographic image obtained at the time of a postoperative increase in the serum total bilirubin concentration. The common bile duct is dilated with intraluminal echogenic material that is likely retained debris.

The present study identified several complications. During IOC, one dog developed suspected contrast‐induced shock characterized by a sudden, marked decrease in blood pressure and discoloration of the intestines, which are findings highly suggestive of an anaphylaxis‐like reaction. In dogs, hemodynamic fluctuations after intravascular administration of iohexol have been reported.^27^ When performing IOC, the possibility of an anaphylaxis‐like reaction with intrabiliary contrast administration should be anticipated preoperatively. In addition, in this series, one dog died after postoperative acute kidney injury, and some dogs developed postoperative jaundice suggestive of acute hepatic dysfunction. Although the causes of these events were unclear and a direct relationship with contrast administration could not be established, iodinated contrast media have been associated with kidney injury in dogs.^28^ In the present study, contrast was administrated intrabiliary rather than intravascularly; however, potential contrast‐related organ injury should also be considered when interpreting postoperative complications after IOC.

Catheter perforation occurred in one dog and should be recognized as a possible technical complication of IOC or BDF. In this case, the cystic duct appeared grossly necrotic, and perforation occurred at that site during catheterization, suggesting that particular caution is warranted when gross abnormalities of the duct are present. Previous canine reports have described advancing the catheter only until the side hole was no longer visible,^16^ suggesting that a practical limit for catheter insertion may be necessary to reduce the risk of iatrogenic injury. In addition, perforation may occur in distal portions of the bile duct that cannot be adequately assessed visually. Therefore, postoperative monitoring with active drainage may be important when bile duct injury is suspected.

Pancreatitis is a common comorbidity in gallbladder disease. Furthermore, there is a reported association between BDF via CBD catheterization during cholecystectomy and postoperative pancreatitis in dogs, with retrograde saline flow into the pancreatic duct proposed as a possible cause.^15^ In the present series, 13 dogs developed postoperative pancreatitis. However, the IOC images did not show findings suggestive of reflux into the pancreatic duct. Thus, in this cohort, IOC findings were not clearly associated with postoperative pancreatitis. Furthermore, a prior study in which BDF and IOC were performed did not identify postoperative pancreatitis.^16^ The mechanism by which pancreatitis occurs after cholecystectomy warrants further investigation.

Antimicrobial use in the present study should be interpreted in the context of the potential clinical impact of biliary infection in dogs undergoing cholecystectomy. Positive biliary culture has been associated with decreased short‐term survival after cholecystectomy for gallbladder mucocele, and biliary culture with empirical preoperative antimicrobial therapy has been recommended in this setting.^29^ In the present study, bacterial growth was detected in 15 dogs (22.1%). Therefore, perioperative antimicrobial use was considered clinically justified, particularly before culture results were available and in dogs with suspected biliary infection, gallbladder rupture, peritoneal effusion, or systemic illness. However, empirical antimicrobial therapy should be accompanied by culture testing whenever possible, and antimicrobial treatment should be reassessed once culture and susceptibility results are available to permit adjustment or de‐escalation of treatment.

The present study had several limitations. First, the procedures were performed by four surgeons, making it difficult to standardize techniques such as BDF. Second, because this was a retrospective study, some examination records were missing. In addition, because undiluted contrast medium was used, visualization of radiolucent or low‐radiopacity calculi may have been limited. Finally, susceptibility data were not uniformly available in a format that allowed a detailed retrospective assessment of antimicrobial appropriateness. Therefore, although bacterial culture results were summarized, the appropriateness of antimicrobial selection, continuation, or de‐escalation could not be fully evaluated. Future investigations should be designed to address these limitations.

## 5 CONCLUSION

During cholecystectomy for benign gallbladder disease in dogs, IOC permits assessment of the CBD and hepatic ducts and is therefore a useful technique when CBD obstruction or retained debris within the CBD or hepatic ducts is suspected. In this study, retained debris was also identified within the hepatic ducts. Because such material may precipitate postoperative CBD obstruction, dogs with retained debris in the hepatic ducts require close monitoring. Procedure‐related complications should also be carefully considered.

## AUTHOR CONTRIBUTIONS

Konno R, DVM: Identified and extracted data from the medical records and drafted and revised the manuscript. Kaneko Y, DVM, PhD: Contributed to the study design, supported the preparation and editing of the final manuscript, was responsible for surgical case management, and provided patient data, including intraoperative details. Matsunaga M, DVM: Extracted and analyzed the data and drafted the initial version of the manuscript. Osuga T, DVM, PhD: Contributed to the study design and supported the preparation and editing of the final manuscript. Okadera R, DVM, Torisu S, DVM, PhD and Yamamoto S, DVM, PhD: Managed the surgical cases and provided patient data, including intraoperative details. Naganobu K, DVM, PhD: Provided anesthesia‐related patient data and assisted with manuscript preparation and editing. All authors critically reviewed the manuscript, approved the final version, and agree to be accountable for all aspects of the work.

## FUNDING INFORMATION

This work was supported by JST SPRING (grant no.: JPMJSP2105) and by AMED (grant no.: JP256f0137007).

## CONFLICT OF INTEREST STATEMENT

The authors declare that there are no conflicts of interest related to this report.

## Supporting information


**Data S1.** Supporting information.

**Video S1.** Catheterization of the cystic duct stump

After cholecystectomy, a catheter was inserted into the cystic duct stump, and a tourniquet was re‐tightened around the stump. Intraoperative cholangiography and bile duct flushing were then performed.

## Data Availability

The data that support the findings of this study are available from the corresponding author upon reasonable request.
