# Supplementary material for: Intraoperative cholangiography during open cholecystectomy in 73 dogs with benign gallbladder disease
Source: Vet Surg. 2026 Sep 9;55(8):1398–408. doi: 10.1111/vsu.70157 (PMC13736187; doi:10.1111/vsu.70157)
Supplement: Supplementary file 1 [file VSU-55-1398-s002.docx]

***Veterinary Surgery* – Additional Submission Questions**

Authors must complete this form and submit it alongside your manuscript via [Research Exchange Submission](https://submission.wiley.com/submission/dashboard?siteName=VSU) as **“Supplementary Material Not For Review.”**

| **Please include the following information about your submission:** | |
| --- | --- |
| Manuscript Title | **Intraoperative cholangiography during open cholecystectomy in 73 dogs with benign gallbladder disease** |
| Date of Submission | November 20, 2025 |

| **Please confirm the following:** | | | | |
| --- | --- | --- | --- | --- |
| The studies described in this manuscript were conducted in a manner consistent with the journal’s standards for animal use and participation of human subjects, as described in the [Author Guidelines](https://onlinelibrary.wiley.com/page/journal/1532950x/homepage/forauthors.html#STUDIES). | ☒ | | Yes | |
| **Please answer the following questions:** | | | | |
| The corresponding authors for all published manuscripts are invoiced by ACVS for page charge fees (see [Author Guidelines](https://onlinelibrary.wiley.com/page/journal/1532950x/homepage/forauthors.html)). For this manuscript, what is the corresponding author's telephone number? | **+81 985-58-7286** | | | |
| All co-authors have completed the [ICMJE’s conflict-of-interest form](https://www.icmje.org/about-icmje/faqs/conflict-of-interest-disclosure-forms/) and returned the information to the corresponding author. Did any author declare interests? | ☐ | Yes | ☒ | No |
| If yes, please state the author and nature of the interest, including any involvement with products and/or company. | | | | |
|  | | | | |

**If applicable, please answer the following questions.**

| This manuscript is submitted by a resident (first author) in consideration for the publication requirement for their ACVS or ECVS credentials. | |
| --- | --- |
| ☐ | Yes, for ACVS credentials. |
| ☐ | Yes, for ECVS credentials. |
| If so, indicate the intended deadline for notification of manuscript acceptance? | |

| Is this manuscript based on research that was funded by the ACVS Foundation? | |
| --- | --- |
| ☐ | Yes, the manuscript contains research funded by the ACVS Foundation. |

**Consideration for an Outstanding Surgery Residents’ Publication Award**

The paper must have been completed by the first author during an ACVS- or ECVS-registered residency training program, and it must be submitted no later than 12 months after completion of the residency program. Only the following article types will be considered: Clinical Research and Experimental Lab Research.

**To be considered for the Outstanding Surgery Residents’ Publication Award, you must complete the online form:** [**www.acvs.org/journal/resident-awards**](http://www.acvs.org/journal/resident-awards)
